# Differentiation of *Hedyotis diffusa* and Common Adulterants Based on Chloroplast Genome Sequencing and DNA Barcoding Markers

**DOI:** 10.3390/plants10010161

**Published:** 2021-01-15

**Authors:** Mavis Hong-Yu Yik, Bobby Lim-Ho Kong, Tin-Yan Siu, David Tai-Wai Lau, Hui Cao, Pang-Chui Shaw

**Affiliations:** 1 Li Dak Sum Yip Yio Chin R & D Center for Chinese Medicine, The Chinese University of Hong Kong, Shatin, N.T., Hong Kong, China; mavisyik@cuhk.edu.hk (M.H.-Y.Y.); 1155047596@link.cuhk.edu.hk (B.L.-H.K.); lautaiwai@cuhk.edu.hk (D.T.-W.L.); 2 Shiu-Ying Hu Herbarium, School of Life Sciences, The Chinese University of Hong Kong, Shatin, N.T., Hong Kong, China; joycesiu@cuhk.edu.hk; 3 Research Center for Traditional Chinese Medicine of Lingnan (Southern China) and College of Pharmacy, Jinan University, Guangzhou 510632, China; kovhuicao@aliyun.com; 4 State Key Laboratory of Research on Bioactivities and Clinical Applications of Medicinal Plants (CUHK), The Chinese University of Hong Kong, Shatin, N.T., Hong Kong, China

**Keywords:** *Hedyotis*, chloroplast genome, herbal medicine, authentication, DNA barcode, ndhD, ycf1, rps16-trnQ

## Abstract

Chinese herbal tea, also known as Liang Cha or cooling beverage, is popular in South China. It is regarded as a quick-fix remedy to relieve minor health problems. *Hedyotis diffusa* Willd. (colloquially Baihuasheshecao) is a common ingredient of cooling beverages. *H. diffusa* is also used to treat cancer and bacterial infections. Owing to the high demand for *H. diffusa*, two common adulterants, *Hedyotis brachypoda* (DC.) Sivar and Biju (colloquially Nidingjingcao) and *Hedyotis corymbosa* (L.) Lam. (colloquially Shuixiancao), are commonly encountered in the market. Owing to the close similarity of their morphological characteristics, it is difficult to differentiate them. Here, we sequenced the complete chloroplast genomes of the three species of *Hedyotis* using next-generation sequencing (NGS). By comparing the complete chloroplast genomes, we found that they are closely related in the subfamily Rubioideae. We also discovered that there are significant differences in the number and repeating motifs of microsatellites and complex repeats and revealed three divergent hotspots, *rps16-trnQ* intergenic spacer, *ndhD* and *ycf1*. By using these species-specific sequences, we propose new DNA barcoding markers for the authentication of *H. diffusa* and its two common adulterants.

## 1. Introduction

Chinese herbal tea, also known as Liang Cha or cooling beverage, is a decoction of multiple herbs popular in South China and tropical Asia [1]. It is used to treat minor illnesses, including flu symptoms. Owing to its popularity, herbal tea was inscribed into the first National List of Intangible Cultural Heritage in 2006 by the State Council of the People’s Republic of China.

Species of *Hedyotis*, a genus of approximately 500 species in the family Rubiaceae [2], are annual or perennial herbs that are mainly distributed in tropical and subtropical regions [3]. Many species of *Hedyotis*, for example, *H. biflora* [4], *H. herbacea* [5] and *H*. *chrysotricha* [6], are used as folk medicine or as ingredients in herbal products for health maintenance. *H. diffusa* is a well-known ingredient in cooling beverages, which are consumed daily to quench thirst and to modulate the immune system [7], to prevent inflammatory diseases and to maintain good health. *H. diffusa* also possesses the pharmacological effects of anti-oxidation, anti-tumor and anti-inflammatory properties for treating different kinds of cancers, such as colorectal cancer, leukemia and multiple myeloma [8,9,10].

The surging demand for *H. diffusa* has resulted in the emergence of two common adulterants, *H. brachypoda* (DC.) Sivar and Biju (colloquially Nidingjingcao) and *H. corymbosa* (L.) Lam. (colloquially Shuixiancao) in the market. Although the morphological characteristics of these three species are only slightly different, they have distinct phytochemicals, for example, Hedyotiscone A is found only in *H. corymbosa* [11] and, 6-O-(E)-p-coumaroyl scandoside methyl ester and 10(S)-hydroxypheophytin only in *H. diffusa* [12]. There have been studies on the authentication of *H. diffusa* using distinctive chemical markers. However, the use of chloroplast genomes for comparing this species with its common adulterants have not been available. To further enhance the quality assurance and quality control of *Hedytois* species in the herbal industry, we set forth to sequence the complete chloroplast genomes of these three species of *Hedyotis*. Besides analyzing their genome organization and features for understanding their phylogenetic relationship, we also found DNA barcoding markers for authenticating them effectively.

## 2. Results

### 2.1. Chloroplast Genome Organization

**Figure 1:**
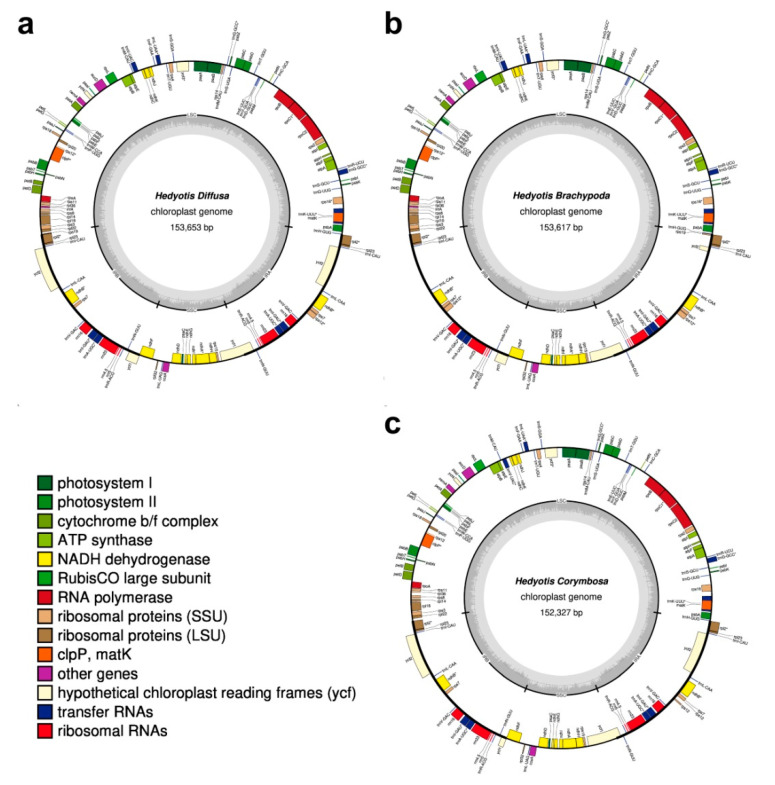
Gene maps of the chloroplast genomes of the three *Hedyotis* species. (**a**) *Hedyotis diffusa*; (**b**) *Hedyotis brachypoda*; (**c**) *Hedyotis corymbosa*. The genes shown inside and outside of the circles are transcribed clockwise and counterclockwise, respectively. Genes belonging to different functional groups are color-coded. The darker gray area and the lighter gray area in the inner circle represent the GC and AT content of the chloroplast genome.

For the three species of *Hedyotis*, paired-end reads with 150 bp in average length were generated by Illumina sequencing. In Genbank, the only available *Hedyotis* chloroplast genome is *Hedyotis ovata* (MK203877). It was used as reference for mapping reads. The sizes of chloroplast genomes of *H. diffusa*, *H. brachypoda* and *H. corymbosa* were 153,653, 153,617 and 152,327 bp, respectively (Figure 1, Table 1). They had a typical quadripartite structure including one large single-copy region (LSC) (83,609 to 83,863 bp), one small single-copy region (SSC) (17,195 to 18,146 bp), and a pair of inverted repeat regions (IRa and IRb) (25,286 to 26,321 bp). The GC contents among the three species were similar with a value around 37%.

Apart from the basic characteristics of the three chloroplast genomes, the genes in the chloroplast genomes were analyzed. The total number of genes encoded in *H. diffusa*, *H. brachypoda* and *H. corymbosa* was 130, 129 and 128, respectively. The encoded genes were classified into four functional categories: protein synthesis and DNA-replication, photosynthesis, miscellaneous group and unknown functions. Genes were classified into different gene groups of the categories (Table 2). The genes containing introns and pseudogenes were analyzed. In addition, there were 19 genes located within the IR regions and therefore were duplicated in the genome.

### 2.2. Analysis of Repeat Sequences

**Figure 2:**
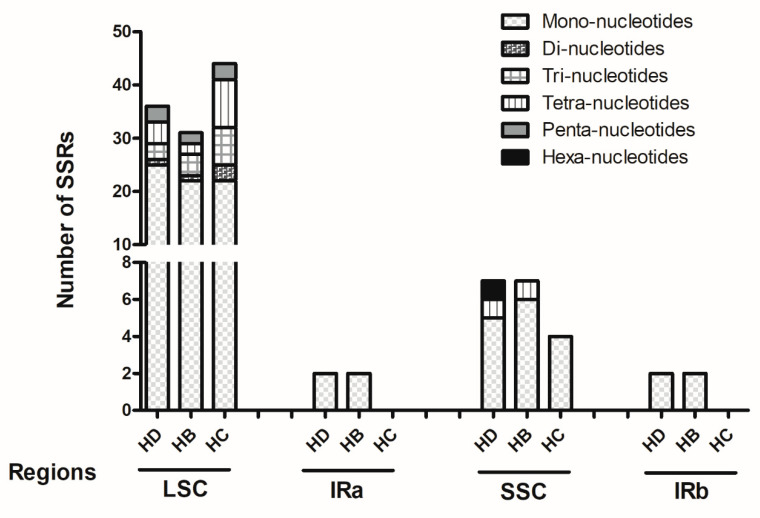
Analysis of simple sequence repeats (SSRs) in the six categories of SSRs in four different regions of the chloroplast genomes. HD: *H. diffusa*, HB: *H. brachypoda*, HC: *H. corymbosa*.

There are variations among the three species of *Hedyotis* in the number of simple sequence repeats and dispersed repeats. The number of simple sequence repeats (SSRs) in *H. diffusa*, *H. brachypoda* and *H. corymbosa* was 47, 42 and 48 respectively. The number of mono-, di-, tri-, tetra-, penta-, and hexa-nucleotides were analyzed (Figure 2). Mono-nucleotide repeats were the most abundant. They accounted for over 50% of the total SSRs, while hexa-nucleotides were the rarest, with only one repeat in *H. diffusa* but none in the others.

**Figure 3:**
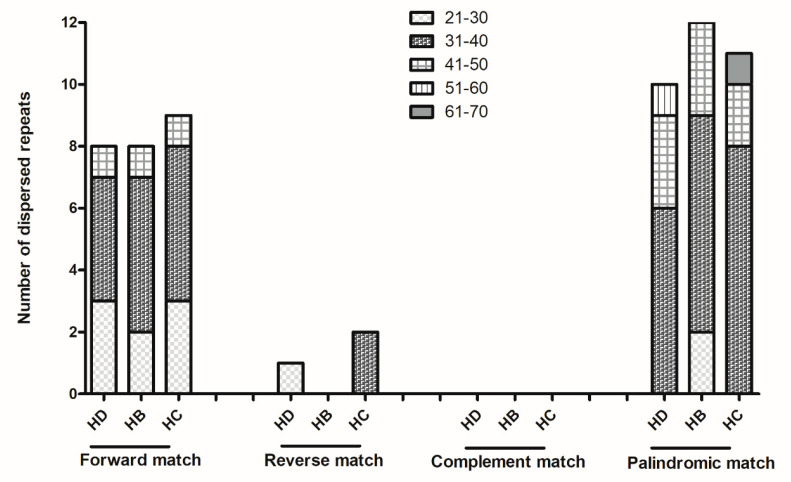
Analysis of four kinds of complex sequence repeats in the three chloroplast genomes. HD: *H. diffusa*, HB: *H. brachypoda*, HC: *H. corymbosa.*

The number of dispersed repeats in *H. diffusa*, *H. brachypoda* and *H. corymbosa* was 19, 20 and 22 respectively. Forward match, reverse match, complement match and palindromic match with repeat length ranging from 21 to 70 bp were analyzed (Figure 3). Most occurring repeats were palindromic matches, followed by forward match, reverse match and complement match.

### 2.3. Genome Sequence Divergence Analysis

**Figure 4:**
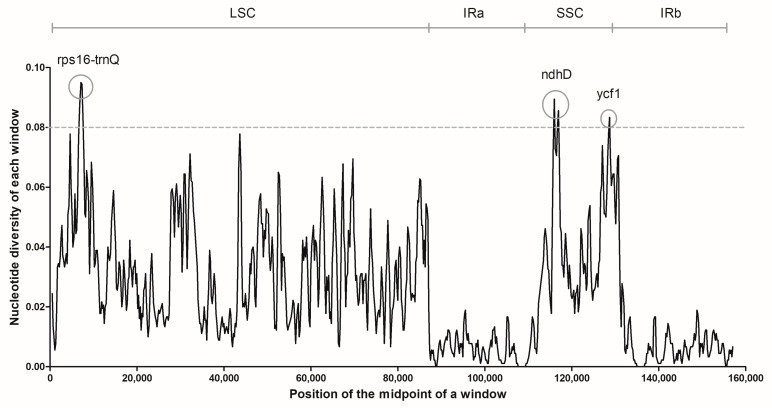
Sliding window analysis of the three chloroplast genomes. Analytic parameters: 600 bp of window length and 200 bp of step size. *X*-axis: Midpoint of a window; *Y*-axis: Nucleotide diversity of each window (Pi).

Nucleotide diversity in the chloroplast genome and highly variable regions can be revealed through sliding window analysis [13]. Comparing the three species of *Hedyotis*, the nucleotide diversity values (Pi) ranged from 0 to 0.095, with an average value of 0.00246. Obviously, sequence divergence was concentrated in the LSC and SSC regions, but showed less variability in the IR regions (Figure 4). Three divergent hotspots *rps16-trnQ* intergenic spacer, *ndhD* and *ycf1*, with distinctive high Pi values of 0.095, 0.089 and 0.083 respectively were revealed. The *rps16-trnQ* intergenic spacer is located in the LSC region, while *ndhD* and *ycf1* are in the SSC and IRb regions respectively.

Insertions/deletions (indels) and single nucleotide polymorphism (SNPs) were also considered (Table 3) for further analyzing the genome sequence divergence. Using the *H. diffusa* chloroplast genome as reference, indels and SNP of the other two chloroplast genomes were compared. The number of SNP and indels of *H. corymbosa* were around 4 and 3 times higher than in *H. brachypoda* when compared to *H. diffusa*, indicating that the genomic difference of *H. corymbosa* is much greater than *H. brachypoda* when compared with *H. diffusa.*

### 2.4. Phylogenetic Analysis

**Figure 5:**
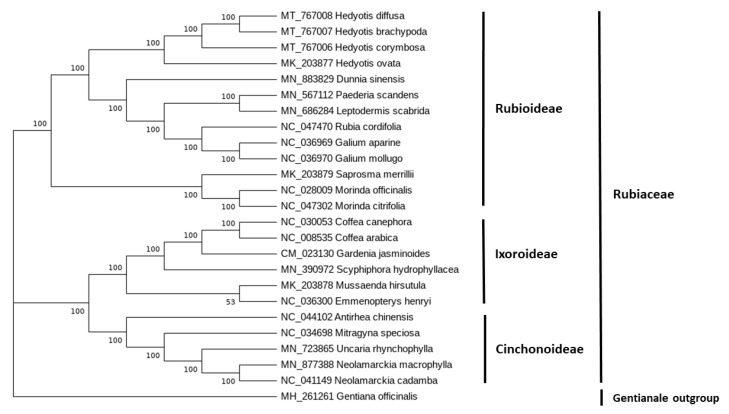
Phylogenetic relationships of the four *Hedyotis* species and other species of Rubiaceae with available complete chloroplast genome sequences using maximum likelihood (ML).

The phylogenetic relationship among plant lineages can be elucidated through the analysis of chloroplast genome sequences [14,15]. Figure 5 shows the phylogenetic relationship of the four species of *Hedyotis* and other species of Rubiaceae. The tree revealed that *H. diffusa* is closer to *H. brachypoda* than *H. corymbosa*. Nevertheless, they all belong in the subfamily of Rubioideae. The phylogenetic tree was highly supported with bootstrap values of 100.

### 2.5. Development of DNA Barcoding Markers

Owing to the short amplicon size and the presence of common region in the *ndhD* and *rps16-trnQ* intergenic spacer among the three *Hedyotis* species, primers were designed for species identification. The *ycf1* region was not chosen as the barcoding region as it is too long and too variable to permit the design of universal primers. This phenomenon for *ycf1* has also been found in *Nicotiana tabacum* [16]. PCR was first performed by using the designed primers on the ten samples. Amplicons were then sequenced, and DNA sequences were aligned with the corresponding regions in the chloroplast genomes we have obtained. Ten samples of *Hedyotis* species were obtained from the market for molecular authentication. Their identities were also identified by morphological characterization. Both approaches give consistent authentication results (Table 4). Sequences of the primers and the amplicons are shown in Tables S1 and S2.

## 3. Discussion

### 3.1. Chloroplast Genome Features

Chloroplasts are vital cell organelles in plants, playing an important role in energy production in photosynthesis and in plant growth and development [17,18]. Same as nucleus and mitochondria, chloroplast has its own genetic material. The basal genomic information helps in addressing phylogenetic and authentication problems [19].

Among the three *Hedyotis* species, the number of protein-coding genes differed, while the number of tRNA and rRNA genes, gene order and clusters were conserved, perhaps due to long-term evolution under environmental pressures, similar to the case of *Robinia* [20]. Genomic differences also derived from the presence of different pseudogenes. *ycf1* is a common one, and its presence may be due to the incomplete duplication of the normal copy between the SSC and IRb regions [21]. There are three other pseudogenes, *rps19* and *ycf2* in *H. brachypoda* and *rps19* and *infA* in *H. corymbosa*. Their existence is due to the occurrence of internal stop codons and results in a massive deletion. These pseudogenes have also been found in other plants, for example, *ycf2* in maize [22], *infA* in *Oenothera elata* [23] and *rps19* in *Malpighiaceae* [24]. In addition, the start codon of the *ndhD* gene, which functions in photosynthesis, had been altered from the common initiation codon AUG to ACG. This may be due to a post transcriptional RNA editing process that induced substitution or indel mutation [25]. The C-to-U RNA editing in plant chloroplasts is common in flowering plants [26].

Repeat sequences contribute to genomic rearrangement, recombination, and sequence divergence [27]. In this study, *H. corymbosa* had the largest number of repeats (70), including both simple sequence repeats and dispersed repeats, while *H. brachypoda* had the fewest (62). The analysis indicated that the genetic variation and diversity may mainly be due to (1) simple sequence repeats—mainly mono-nucleotides, but also hexa-nucleotides—and (2) dispersed repeats: palindromic match with 31–40 repeat lengths. Moreover, over 70% of the simple repeats were located in the LSC region, followed by the SSC region and the IR region, suggesting that the IR regions are less variable than the other two regions [28].

### 3.2. Sequence Divergence, Phylogenetic Relationships and Molecular Markers for Authentication

The sequence divergence was clearly indicated by the sliding window and indels/SNPs analysis. The nucleotide diversity in IR regions is low, with Pi values less than 0.02, which means less genetic variation among the three species of *Hedyotis* in the IR regions. This phenomenon was consistent with findings in other chloroplast genome studies [29,30], which suggests the low sequence variation in IR regions was by virtue of gene conversion for copy correction of IR sequences [31]. The three hotspot regions discovered with Pi values higher than 0.08 have potential to develop DNA barcoding markers [16]. Moreover, the indels/SNPs results revealed that the genomic difference of *H. corymbosa* is much higher than *H. brachypoda* when compared with *H. diffusa.* This showed a consistent conclusion with the phylogenetic relationship of the three species of *Hedyotis*.

With the genetic variation discovered, we analyzed the phylogenetic relationship of the four species of *Hedyotis* and other species of Rubiaceae using complete chloroplast genomes for the determination of their phylogenetic relationship. From the phylogenetic analysis, all the Rubiaceae species were basically divided into two branches. One branch consisted of species in the subfamily of Rubioideae, while another consisted of species in subfamilies of Ixoroideae and Cinchonoideae. All four species of *Hedyotis* belonged to the subfamily of Rubioideae. The analytical result revealed that *H. diffusa* and *H. brachypoda* are closer to each other with the same branching point; however, they are less close to *H. corymbosa* as well as *H. ovata*. The phylogenetic analysis is consistent with the classification of *Flora Republicae Popularis Sinicae*. Both the *H. diffusa* and *H. corymbosa* belong to the section Euoldenlandia, while *H. ovata* belongs to section Diplophragma. According to *Flora of China*, *H. brachypoda* is commonly circumscribed as *H. diffusa*, although the taxonomy of these two species is unsolved. As expected, *H. diffusa* and *H. brachypoda* had a closer relationship in our phylogenetic analysis. *H. ovata* was also successfully classified into a different lineage with the other three *Hedyotis* species.

Recently, the use of whole chloroplast genomes in developing specific barcodes for distinguishing closely related plant species has been proposed [32,33]. DNA barcoding is a widely used tool for species identification and authentication [34]. It has also been used to identify material present in processed seafood products for promoting food safety [35]. In this study, we designed primers with amplicon size 200–300 bp from the two identified hotspot regions *ndhD* and *rps16-trnQ* intergenic spacer. DNA from dried herbs are always degraded during manufacturing processes [36]. The proposed specific barcodes are superior to universal barcodes because (1) the specific primers are designed from hotspot regions with great variations among target species, which is good for identification, and (2) the amplicons are small enough, which is more appropriate for processed herbal products. With the specific barcodes, the quality control of *Hedyotis*-containing herbal beverages can be enhanced.

## 4. Materials and Methods

### 4.1. Collection of Plant Samples

Fresh plants of *H. diffusa*, *H. brachypoda and H. corymbosa* were collected at various locations in the Chinese University of Hong Kong. The samples with voucher specimens were deposited in the Shiu-Ying Hu Herbarium, Chinese University of Hong Kong. The specimens were collected based on the delimitation of the three species according to the treatment of Sivarajan and Biju [37]. The identification of *H. diffusa* was based on the taxonomic treatment characterizing species with 3 to 7 flowered cymes or 1 to 2 flowered cymes and without a ring of hairs in the corolla. *H. brachypoda* was characterized as being 1 to 2 flowered and lacking a ring of hairs in the corolla. *H. corymbosa* was characterized by having 3 to 8 flowered cymes or rarely with solitary flower, and with a ring of hairs in the corolla.

### 4.2. DNA Extraction

For DNA extraction, 200 mg of fresh plant samples were ground by liquid nitrogen for DNA extraction using DNeasy plant mini kit (Qiagen, Germany), and 400 μL AP1 extraction buffer, 4% Polyvinylpyrrolidone (PVPP), 2% β-mercaptoethanol and 4 μL RNase A (100 mg/mL) were added to the samples and incubated at 65 °C for 10 min. The protocol of the manufacturer was then followed. Fifty microliters elution buffer was used to collect the purified DNA.

### 4.3. Chloroplast Genome Sequencing, Assembling and Annotation

The three samples of extracted genomic DNA were sequenced by Novogene Corporation Inc., China. Libraries were generated using NEBNext DNA Library Prep Kit (NEB, Ipswich, MA, USA) and sequenced using the Illumina NovaSeq 6000 platform (Illumina, USA). The number of pair-end reads of *H. diffusa*, *H. brachypoda* and *H. corymbosa* were 169,465, 174,248 and 169,635 respectively, with 150 bp read length. The raw data were trimmed and assembled into contigs using CLC assembly cell v4.21.104315 and SOAPdenovo v. 3.23, with default parameters set. Gapcloser module in SOAP package was used for gap filling. A reference genome of *H. ovata* (MK203877) was downloaded for contigs alignment and chloroplast genome assembly. All paired-end reads were mapped to the assembled chloroplast genomes with over 150 × coverage. Two other reference genomes, *Galium aparine* (NC036969) and *Galium mollugo* (NC036970), were also downloaded for gene annotation using the Geseq platform. Annotated genes were manually verified and adjusted. The circular plastid genome maps were then drawn using OrganellarGenomeDRAW [38] (Figure 1). The three annotated chloroplast genomes were deposited in GenBank (https://www.ncbi.nlm.nih.gov) with corresponding accession numbers.

### 4.4. Repeat Elements Analysis

MISA PERL script [39] was used to detect the SSRs in the chloroplast genomes. For the SSR search parameters, the minimum number of repetitions of mono-, di-, tri-, tetra-, penta-, and hexa-nucleotides was 10, 5, 4, 3, 3 and 3, respectively. REPuter [40] was used to detect the size and location of repeats in the genome sequences. Four types of repeats: forward match, reverse match, complement match and palindromic match, were searched and analyzed. Parameters were set as three of hamming distance, 100 of maximum computed repeats and 30 of minimal repeat size.

### 4.5. Sequence Divergence Analysis

The chloroplast genomic sequences of the three species of *Hedyotis* were aligned by MAFFT [41]. DnaSP (DNA Sequences Polymorphism v6.12.03) [42] was used to analyze the nucleotide polymorphisms from the three aligned DNA sequences. The parameters for sliding window analysis were set as 600 sites of window length and 200 sites of step size. Nucleotide diversity (Pi) of each mid-point was computed for constructing a DNAsp graph indicating the sequence variation among the three species of *Hedyotis* (Figure 4). In-house python coding developed by Seoul National University was used to determine the number and position of indels and SNPs.

### 4.6. Phylogenetic Analysis

Phylogenetic analysis was conducted using the complete chloroplast genomic sequences of the three *Hedyotis* species in this study and 21 species of Rubiaceae available in NCBI, including *Hedyotis ovata* (MK203877), *Leptodermis scabrida* (MN686284), *Paederia scandens* (MN567112), *Dunnia sinensis* (MN883829), *Rubia cordifolia* (NC047470), *Galium mollugo* (NC036970), *Galium aparine* (NC036969), *Saprosma merrillii* (MK203879), *Morinda officinalis* (NC028009), *Morinda citrifolia* (NC047302), *Coffea arabica* (NC008535), *Coffea canephora* (NC030053), *Gardenia jasminoides* (CM023130), *Scyphiphora hydrophyllacea* (MN390972), *Emmenopterys henryi* (NC036300), *Mussaenda hirsutula* (MK203878), *Antirhea chinensis* (NC_044102), *Mitragyna speciosa* (NC034698), *Uncaria rhynchophylla* (MN723865), *Neolamarckia cadamba* (NC041149) and *Neolamarckia macrophylla* (MN877388). *Gentiana officinalis* (MH261261) was used as an outgroup. The complete chloroplast genomic sequences were aligned by MAFFT [41]. The best nucleotide substitution model (GTR + G +I) was tested. Mega-X software [43] was used to construct the maximum likelihood (ML) with 1000 bootstrap replicates.

### 4.7. Development of DNA Barcoding Markers

The genomic sequences of the three *Hedyotis* species were first aligned by MAFFT. Primers were designed (Table S1) from the hotspot divergence regions concluded from the sequence divergence analysis session. The designed primers were subjected to OligoAnalyzer 3.1 (Integrated DNA Technologies, Inc., Coralville, IA, USA) for evaluation. Ten *Hedyotis* samples were collected from herbal shops in Hong Kong. DNA was extracted and amplified by polymerase chain reaction (PCR). The PCR products were purified and sequenced by Sanger sequencing (BGI, Hong Kong, China). Sequenced amplicons were aligned with our chloroplast genomes by MAFFT.

## 5. Conclusions

In this study, the complete chloroplast genomes of *H. diffusa*, *H. brachypoda* and *H. corymbosa* were constructed and analyzed. The genome size ranged from 152,327 bp to 153,653 bp. Different comparative analyses showed that the genomic differences were derived from: (1) the number of protein-coding genes, (2) the presence of various pseudogenes, (3) the number and distribution of simple and dispersed repeats and (4) the distinct species-specific sequences. These findings enhanced our knowledge and understanding of the chloroplast genomes and the phylogenetic relationship of the four *Hedyotis* species and other species of Rubiaceae. The phylogenetic tree indicated that the four species of *Hedyotis* were closely related. This study also developed DNA barcoding markers from *ndhD* and *rps16-trnQ* for species authentication. Proper authentication of herbal material is of the utmost importance. The correct use of herbal ingredients in cooling beverages helps to enhance quality assurance and quality control in the herbal industry and safeguard the safety of consumers. We anticipate that the two unique DNA markers generated in this study can be used for quality control and authentication of *H. diffusa* in the herbal industry.

## Figures and Tables

**Table 1:** The basic characteristics of the chloroplast genomes of the three *Hedyotis* species.

Characteristics	*Hedyotis diffusa* (MT767008)	*Hedyotis brachypoda* (MT767007)	*Hedyotis corymbosa* (MT767006)
**Specimen Voucher**	H2200	H2174	H2106
**Total Size (bp)**	153,653	153,617	152,327
**Large Single Copy (LSC, bp)**	83,863	83,780	83,609
**Small Single Copy (SSC, bp)**	17,218	17,195	18,146
**Inverted Repeat (IR, bp)**	26,286	26,321	25,286
**Total number of genes**	130	129	128
**Protein-coding genes**	85	84	83
**tRNA genes**	37	37	37
**rRNA genes**	8	8	8
**GC content (%)**	37.62	37.61	37.41

**Table 2:** List of genes in the chloroplast genomes of the three *Hedyotis* species.

Category	Gene Group	Gene Name
Protein synthesis and DNA-replication	Ribosomal RNA genes	*rrn4.5, rrn5, rrn16, rrn23*
	Transfer RNA genes	trnH-GUG, trnL-UAG, trnQ-UUG, trnS-GCU, trnR-UCU, trnC-GCA, trnD-GUC, trnY-GUA, trnE-UUC, trnT-GGU, trnS-UGA, trnfM-CAU, trnS-GGA, trnT-UGU, trnF-GAA, trnM-CAU, trnW-CCA, trnP-UGG, trnV-UAC, trnL-UAA *, trnK-UUU *, trnG-GCC *, **trnI-CAU**, **trnL-CAA**, **trnV-GAC**, **trnI-GAU** *, **trnA-UGC** *, **trnR-ACG**, **trnN-GUU**
	Small subunit of ribosome	rps2, rps3, rps4, **rps7**, rps8, rps11, **rps12** *, rps14, rps15, rps16 *, rps18, **rps19**^δ^
	Large subunit of ribosome	**rpl2** *, rpl14, rpl16, rpl20, rpl22, **rpl23**, rpl32, rpl36
	RNA polymerase subunits	rpoA, rpoB, rpoC1 *, rpoC2
Photosynthesis	NADH dehydrogenase	ndhA *, **ndhB** *, ndhC, ndhD, ndhE, ndhF, ndhG, ndhH, ndhI, ndhJ, ndhK
	Photosystem I	psaA, psaB, psaC, psaI, psaJ, ycf3 **
	Photosystem II	*psbA, psbB, psbC, psbD, psbE, psbF, psbH, psbI, psbJ, psbK, psbL, psbM, psbN, psbT, psbZ*
	Cytochrome b/f complex	*petA, petB, petD, petG, petL, petN*
	ATP synthase	*atpA, atpB, atpE, atpF, atpH, atpI*
	Large subunit of rubisco	*rbcL*
Miscellaneous group	Maturase	*matK*
	Protease	clpP **
	Envelope membrane protein	*cemA*
	Subunit of acetyl-CoA-carboxylase	*accD*
	c-type cytochrome synthesis	*ccsA*
	Component of TIC complex	***ycf1**^Ψ^*
	Translation initiation factor	*infA ^ω^*
	Hypothetical chloroplast reading frames	***ycf2**^σ^*
Pseudogene unknown function	ORFs	*ycf4*

* Genes containing one introns; ** Genes containing two introns; ^Ψ^ Pseudogene in *H*. *diffusa*, *H*. *brachypoda* and *H*. *corymbosa*; ^δ^ Pseudogene in *H*. *brachypoda* and *H*. *corymbosa* only; ^σ^ Pseudogene in *H*. *brachypoda* only; ^ω^ Pseudogene in *H*. *corymbosa* only; Genes in **bold** are located within the IR and therefore are duplicated.

**Table 3:** Comparison of insertions/deletions (indels) and single nucleotide polymorphisms (SNPs) of the three *Hedyotis species*. The upper and lower triangles show the number of SNPs and indels in the complete chloroplast genomes respectively.

	*Hedyotis diffusa*	*Hedyotis brachypoda*	*Hedyotis corymbosa*
***Hedyotis diffusa***		1051	4996
***Hedyotis brachypoda***	198		5061
***Hedyotis corymbosa***	684	677	

**Table 4:** Molecular authentication of *Hedyotis* samples from the market.

Specimen Voucher	Percentage Identity with *ndhD* in Chloroplast Genome			Percentage Identity with *rps16-trnQ* in Chloroplast Genome			Sample Identity
	*H. corymbosa*	*H. brachypoda*	*H. diffusa*	*H. corymbosa*	*H. brachypoda*	*H. diffusa*	
T5084	100%	97%	97%	99%	85%	85%	*H. corymbosa*
T5089	97%	100%	99%	86%	97%	96%	*H. brachypoda*
T5093	100%	97%	97%	99%	87%	86%	*H. corymbosa*
T5097	100%	97%	97%	99%	87%	87%	*H. corymbosa*
T5101	97%	100%	99%	87%	98%	97%	*H. brachypoda*
T5106	100%	97%	97%	100%	87%	87%	*H. corymbosa*
T5110	97%	100%	99%	87%	98%	97%	*H. brachypoda*
T5114	97%	100%	99%	87%	98%	97%	*H. brachypoda*
T5121	97%	100%	99%	87%	98%	97%	*H. brachypoda*
T5126	97%	100%	99%	87%	98%	97%	*H. brachypoda*

## Data Availability

The chloroplast genomes presented in this study are openly available in GenBank with reference number MT767006-MT767008.

## Supplementary Materials

The following are available online at https://www.mdpi.com/2223-7747/10/1/161/s1, Table S1: Sequences and parameters of the designed primers. Table S2: The amplified sequences from the ten sample.
