# Supplementary material for: Differentiation of Hedyotis diffusa and Common Adulterants Based on Chloroplast Genome Sequencing and DNA Barcoding Markers
Source: Plants (Basel). 2021 Jan 15;10(1):161. doi: 10.3390/plants10010161 (PMC7829813; doi:10.3390/plants10010161)
Supplement: Supplementary file 1 [file plants-10-00161-s001.pdf]

Table S1. Sequences and parameters of the designed primers.

	DNA Loci	Primers Name	Primer Sequence (5’ to 3’)	Annealing Temperature (°C)	Amplicon Size (bp)
**1**	*ndhD*	HD_ndhD_S_F	ACCCATACGCTCCCATTTTT	52	200 - 300
		HD_ndhD_LS_R	GGGAGTTTTGGGTATTGCTTTA		
**2**	*rps16-trnQ*	HD_ rps16-trnQ_F2	TCTGTTGACAAAGAGTAATAGA		
		HD_ rps16-trnQ _R2	AAACCCAATCTAGTTCAAAC		

Table S2. The amplified sequences from the ten samples.

DNA Loci	Sample voucher number	Amplified sequences
ndhD	T5084	AATAAGATTCCAGCCAAAAGCATACAAGTACTGTAATGCGCTTCTCCGTGGGTATCCGGTAACCATGTGTGTAGGGGTATAATCGGTGATTTGACAGCAAAAGCAATA<br>AAAAATCCAATATAAAATAGTATTTCCAAGGCTACAGGATAAGACTGATTAGCTGATGTTTCAAAATTTAATGTTGGTTCAGCGGAACCATA
	T5089	AATAAGATTCCGGCCAAAAGCATACAAGTACTATAATGTGCTTCTCCGTGGGTATCCGGTAACCATGTGTGTAGGGGTATAATCGGCGATTTGACAGCAAAAGCAATAA<br>AAAATCCAATATAGAATAGTATTTCCAAGGCTACAGGATAAGACTGATTAGCTGATGTTTCAAAATTTAATGTTGGTTCAGTGGAACCATA
	T5093	AATAAGATTCCAGCCAAAAGCATACAAGTACTGTAATGCGCTTCTCCGTGGGTATCCGGTAACCATGTGTGTAGGGGTATAATCGGTGATTTGACAGCAAAAGCAATAA<br>AAAATCCAATATAAAATAGTATTTCCAAGGCTACAGGATAAGACTGATTAGCTGATGTTTCAAAATTTAATGT-TGGTTCAGCGGAACCATA
	T5097	AATAAGATTCCAGCCAAAAGCATACAAGTACTGTAATGCGCTTCTCCGTGGGTATCCGGTAACCATGTGTGTAGGGGTATAATCGGTGATTTGACAGCAAAAGCAATAA<br>AAAATCCAATATAAAATAGTATTTCCAAGGCTACAGGATAAGACTGATTAGCTGATGTTTCAAAATTTAATGTTGGTTCAGCGGAACCATA
	T5101	AATAAGATTCCGGCCAAAAGCATACAAGTACTATAATGTGCTTCTCCGTGGGTATCCGGTAACCATGTGTGTAGGGGTATAATCGGCGATTTGACAGCAAAAGCAATAA<br>AAAATCCAATATAGAATAGTATTTCCAAGGCTACAGGATAAGACTGATTAGCTGATGTTTCAAAATTTAATGTTGGTTCAGTGGAACCATA
	T5106	AATAAGATTCCAGCCAAAAGCATACAAGTACTGTAATGCGCTTCTCCGTGGGTATCCGGTAACCATGTGTGTAGGGGTATAATCGGTGATTTGACAGCAAAAGCAATA<br>AAAAATCCAATATAAAATAGTATTTCCAAGGCTACAGGATAAGACTGATTAGCTGATGTTTCAAAATTTAATGTTGGTTCAGCGGAACCATA
	T5110	AATAAGATTCCGGCCAAAAGCATACAAGTACTATAATGTGCTTCTCCGTGGGTATCCGGTAACCATGTGTGTAGGGGTATAATCGGCGATTTGACAGCAAAAGCAATAA<br>AAAATCCAATATAGAATAGTATTTCCAAGGCTACAGGATAAGACTGATTAGCTGATGTTTCAAAATTTAATGTTGGTTCAGTGGAACCATA
	T5114	AATAAGATTCCGGCCAAAAGCATACAAGTACTATAATGTGCTTCTCCGTGGGTATCCGGTAACCATGTGTGTAGGGGTATAATCTGGCGATTTGACAGCAAAAGCAATA<br>AAAAATCCAATATAGAATAGTATTTCCAAGGCTACAGGATAAGACTGATTAGCTGATGTTTCAAAATTTAATGTTGGTTCAGTGGAACCATA
	T5121	AATAAGATTCCGGCCAAAAGCATACAAGTACTATAATGTGCTTCTCCGTGGGTATCCGGTAACCATGTGTGTAGGGGTATAATCGGCGATTTGACAGCAAAAGCAATAA

		AAAATCCAATATAGAATAGTATTTCCAAGGCTACAGGATAAGACTGATTAGCTGATGTTTCAAAATTTAATGTTGGTTCAGTGGAACCATA
	T5126	AATAAGATTCCGGCCAAAAGCATACAAGTACTATAATGTGCTTCTCCGTGGGTATCCGGATAACCATGTGTGTAGGGGTATAATCGGCGATTTGACAGCAAAAGCAATAA<br>AAAATCCAATATAGAATAGTATTTCCAAGGCTACAGGATAAGACTGATTAGCTGATGTTTCAAAATTTAATGTTGGTTCAGTGGAACCATA
DNA Loci	Sample voucher number	Amplified sequences
rps16-trnQ	T5084	TAATTAGTAAAAACTCGATTGTTAATAAAATGGAAAATAGATATGAAACAGAAGGTGTTTCCAAATAACTAACTTATCTAAAGGTGTTTCTAAATAACTAACTT<br>ATCTAACCCTAAATACAAAATAGAAAGCATATGATCAACCTTTTTTTTGAATTCAAATTCTCATAATCCATGTTCTCACCTTAACTGGATCCTCAATCCCATTAAA<br>TTACACCCATTT
	T5089	TAATTACTAAAAATTTCATTTTTAATAAAATGGAAAATCGATATCGGAACAAAGTGTTGTTTCCAAATAACAAACTTACTTAACCCTAAATAGAAAGGTCTTGA<br>ACATATGATCAACTTTGTTTTTGAATTCAAATTCTCATAATCCATGTTCCGACCTTAACTGGATCCTTAATTGATTAAATTACACCC
	T5093	TAATTAGTAAAAACTCGATTGTTAATAAAATGGAAAATAGATATGAAACAGAAGGTGTTTCCAAATAACTAACTTATCTAAAGGTAGTTTCTAAATAACTAACTT<br>ATCTAACCCTAAATACAAAATAGAAAGCATATGATCAACCTTTTTTTTGAATTCAAATTCTCATAATCCATGTTCTCACCTTAACTGGATCCTCAATCGATTAAATT<br>ACACCCATTT
	T5097	TAATTAGTAAAAACTCGATTGTTAATAAAATGGAAAATATGATATGAAACAGAAAGGTGTTTCCAAATAACTAACTTATCTAAAGGTGTTTCTAAATAACTAACTT<br>ATCTAACCCTAAATACAAAATATAAAGCATATGATCAACCTTTTTTTTGAATTCAAATTCTCATAATCCATGTTCTCACCTTAACTGGATCCTCAATCGATTAAATTAC<br>ACCCATTT
	T5101	TAATTACTAAAAATTTCATTTTTAATAAAATGGAAAATCGATATGGAACAAAGGTTGTTTCCAAATAACAAACTTACTTAACCCTAAATAGAAAGGTCTTGAACA<br>TATGATCAACTTTGTTTTTGAATTCAAATTCTCATAATCCATGTTCCGACCTTAACTGGATCCTTAATTGATTAAATTACACCC
	T5106	TAATTAGTAAAAACTCGATTGTTAATAAAATGGAAAATAGATATGAAACAGAAGGTGTTTCCAAATAACTAACTTATCTAAAGGTGTTTCTAAATAACTAACTTAT<br>CTAACCCTAAATACAAAATAGAAAGCATATGATCAACCTTTTTTTTGAATTCAAATTCTCATAATCCATGTTCTCACCTTAACTGGATCCTCAATCGATTAAATTACA

		CCCATTTGTTT
	T5110	TAATTACTAAAAATTTCATTTTTAATAAAATGGAAAATCGATATGGAACAAAGGTTGTTTCCAAATAACAAACTTACTTAACCCTAAATAGAAAGGTCTTGAACAT<br>ATGATCAACTTTGTTTTTGAATTCAAATTCTCATAATCCATGTTCCGACCTTAACTGGATCCTTAATTGATTAAATTACACCC
	T5114	TAATTACTAAAAATTTCATTTTTAATAAAATGGAAAATCGATATGGAACAAAGGTTGTTTCCAAATAACAAACTTACTTAACCCTAAATAGAAAGGTCTTGAACAT<br>ATGATCAACTTTGTTTTTGAATTCAAATTCTCATAATCCATGTTCCGACCTTAACTGGATCCTTAATTGATTAAATTACACCC
	T5121	TAATTACTAAAAATTTCATTTTTAATAAAATGGAAAATCGATATGGAACAAAGGTTGTTTCCAAATAACAAACTTACTTAACCCTAAATAGAAAGGTCTTGAACATAT<br>GATCAACTTTGTTTTTGAATTCAAATTCTCATAATCCATGTTCCGACCTTAACTGGATCCTTAATTGATTAAATTACACCC
	T5126	TAATTACTAAAAATTTCATTTTTAATAAAATGGAAAATCGATATGGAACAAAGGTTGTTTCCAAATAACAAACTTACTTAACCCTAAATAGAAAGGTCTTGAACATAT<br>GATCAACTTTGTTTTTGAATTCAAATTCTCATAATCCATGTTCCGACCTTAACTGGATCCTTAATTGATTAAATTACACCC
